# Pandemic and post-pandemic Influenza A (H1N1) infection in critically ill patients

**DOI:** 10.1186/cc10573

**Published:** 2011-11-28

**Authors:** Ignacio Martin-Loeches, Emili Díaz, Loreto Vidaur, Antoni Torres, Cesar Laborda, Rosa Granada, Juan Bonastre, Mar Martín, Josu Insausti, Angel Arenzana, Jose Eugenio Guerrero, Ines Navarrete, Jesus Bermejo-Martin, David Suarez, Alejandro Rodriguez

**Affiliations:** 1 Critical Care Center, Parc Taulí Hospital-Sabadell, CIBERes, Sabadell, Spain; 2 Critical Care Department, Hospital Joan XXIII/CIBERES/IISPV/URV- Tarragona, Spain; 3 Critical Care Department, Hospital Donostia/CIBERES - San Sebastian, Spain; 4 Pneumology Department, Hospital Clínic of Barcelona, IDIBAPS, Centro de Investigación Biomédica en Red-Enfermedades Respiratorias (CIBERES, CB06/06/0028), University of Barcelona, Barcelona, Spain; 5 Critical Care Department. Hospital Vall d'Hebron/CIBERES/IRVH - Barcelona, Spain; 6 Critical Care Department, Hospital de Bellvitge/CIBERES - Barcelona, Spain; 7 Critical Care Department, Hospital La Fe -Valencia, Spain; 8 Critical Care Department, Hospital La Candelaria - Tenerife, Spain; 9 Critical Care Department, Hospital de Navarra - Pamplona, Spain; 10 Critical Care Department, Hospital V. de la Macarena/CIBERES - Sevilla, Spain; 11 Critical Care Department, Hospital G.Marañón/CIBERES - Madrid, Spain; 12 Critical Care Department, Hospital V. de las Nieves - Granada, Spain; 13 Infection and Immunity Unit, Hospital Clínico Universitario-IECSCYL, Valladolid, Spain; 14 Epidemiology and Assessment Unit, Fundació Parc Tauli, Universitat Autònoma de Barcelona, Sabadell, Spain

## Abstract

**Background:**

There is a vast amount of information published regarding the impact of 2009 pandemic Influenza A (pH1N1) virus infection. However, a comparison of risk factors and outcome during the 2010-2011 post-pandemic period has not been described.

**Methods:**

A prospective, observational, multi-center study was carried out to evaluate the clinical characteristics and demographics of patients with positive RT-PCR for H1N1 admitted to 148 Spanish intensive care units (ICUs). Data were obtained from the 2009 pandemic and compared to the 2010-2011 post-pandemic period.

**Results:**

Nine hundred and ninety-seven patients with confirmed An/H1N1 infection were included. Six hundred and forty-eight patients affected by 2009 (pH1N1) virus infection and 349 patients affected by the post-pandemic Influenza (H1N1)v infection period were analyzed. Patients during the post-pandemic period were older, had more chronic comorbid conditions and presented with higher severity scores (Acute Physiology And Chronic Health Evaluation II (APACHE II) and Sequential Organ Failure Assessment (SOFA)) on ICU admission. Patients from the post-pandemic Influenza (H1N1)v infection period received empiric antiviral treatment less frequently and with delayed administration. Mortality was significantly higher in the post-pandemic period. Multivariate analysis confirmed that haematological disease, invasive mechanical ventilation and continuous renal replacement therapy were factors independently associated with worse outcome in the two periods. HIV was the only new variable independently associated with higher ICU mortality during the post-pandemic Influenza (H1N1)v infection period.

**Conclusion:**

Patients from the post-pandemic Influenza (H1N1)v infection period had an unexpectedly higher mortality rate and showed a trend towards affecting a more vulnerable population, in keeping with more typical seasonal viral infection.

## Introduction

There is a vast amount of information published regarding the impact of the 2009 pandemic Influenza A (H1N1)v infection [1,2]. The pandemic represented a challenge for physicians worldwide, manifesting with the acute onset of respiratory failure in a patient population often young, with few comorbid conditions. Several recommendations have been considered, taking into account the literature published during this time. The early use of oseltamivir showed a survival benefit [3,4], while the use of systemic corticosteroids did not [5,6]. Identification of risk factors, such as the presence of community acquired respiratory co-infection (CARC) [7], obesity [8] and the development of acute kidney injury, have helped physicians gain a better understanding of the illness [9].

Health authorities warned that clinical suspicion should be maintained following the initial pandemic, with a post-pandemic period predicted for the 2010-2011 winter as a result of the former A/H1N1 2009 pandemic virus, currently called "new A/H1N1 virus" (An/H1N1) [10].

The aim of the present study was to compare risk factors, clinical features and outcomes in pandemic influenza An/H1N1 patients with those observed in the immediate post-pandemic influenza period.

## Material and methods

This prospective, observational cohort study of intensive care unit (ICU) patients was conducted across 148 ICUs in Spain. Data were obtained from a voluntary registry created by the Spanish Society of Intensive Care Medicine (SEMICYUC), the Spanish Network for Research on Infectious Disease (REIPI) and the Spanish Biomedical Research Center Network on Respiratory Diseases (CIBERES). The study was approved by the Joan XXIII University Hospital Ethics Committee (*IRB NEUMAGRIP/11809*). Patient identification remained anonymous. The requirement for informed consent was waived due to the observational nature of the study and the fact that this activity is an emergency public health response as reported elsewhere [11].

Data were reported by the attending physician after reviewing medical charts and radiological and laboratory records. Two periods were analyzed based on data on all patients within the cohort consecutively diagnosed with An/H1N1 influenza: the 2009 pandemic (H1N1)v infection period between epidemiological weeks 23 and 52 of 2009, and the post-pandemic Influenza (H1N1)v infection period between epidemiological weeks 50 and 52 of 2010 and weeks 1 to 9 of 2011. Children under 15 years old were not enrolled in the registry. The An/H1N1 infection was confirmed by means of real-time reverse-transcription-polymerase chain reaction (RT-PCR) on either nasopharyngeal swab samples or tracheal secretions ordered by the attending physicians at intensive care unit (ICU) admission. An/H1N1 testing was performed in each institution or centralized in a reference laboratory when local resources were not available. RT-PCR methods and further details are described elsewhere [11]. A confirmed case was defined as an acute respiratory illness with laboratory-confirmed An/H1N1. Only confirmed cases were included in the current report.

The ICU admission criteria and treatment decisions for all patients, including determination of the need for intubation and type of antibiotic or antiviral therapy administered, were made at the discretion of the attending physician and not standardized. Septic shock and Multiple Organ Dysfunction Score (MODS) were defined following the criteria of the American College of Chest Physicians and the Society of Critical Care Medicine [12].

Systemic corticosteroid use was implemented when patients developed shock (hydrocortisone), or coadjuvant treatment was used for pneumonia (methylprednisolone). Orally administered oseltamivir (150 mg/24 h or 300 mg/24 h) or intravenous zanamivir (600 mg/12 h) was chosen by the attending physician.

Primary viral pneumonia was defined where patients presented with acute respiratory distress, unequivocal alveolar opacities involving two or more lobes, and negative respiratory and blood bacterial cultures during the acute phase of influenza virus infection. Community-Acquired Respiratory Co-infection (CARC) was defined as any infection diagnosed within the first two days of hospitalization [7]. Infections occurring later were considered nosocomial. hospital-acquired pneumonia (HAP) was defined based on current American Thoracic Society and Infectious Disease Society of America guidelines [13]. Patients who presented with healthcare-associated pneumonia (HCAP) were excluded from the present study [13]. Hematological disease was defined in those patients who presented with acute lymphoblastic leukemia, acute myeloblastic leukemia, chronic lymphocytic leukemia, chronic myelogenous leukemia, Hodgkin's lymphoma, non-Hodgkin's lymphoma, myeloma, graft versus host disease or post-bone marrow transplantation. Obese patients were defined as those with a body mass index (BMI) greater than 30 kg/m^2 ^[8]. Features such as smoking history and immunosuppressive factors were not recorded. Patients who had previously received Influenza A (H1N1) 2009 monovalent or seasonal influenza 2010-2011 vaccination were considered to be "vaccinated". Acute kidney injury (AKI) and its stages were diagnosed according to the glomerular filtration rate (GFR) criteria of the current Acute Kidney Injury Network definitions [14]. Diagnostic criteria for acute kidney injury (AKI): An abrupt (within 48 hours) reduction in kidney function currently defined as an absolute increase in serum creatinine of more than or equal to 0.3 mg/dl, a percentage increase in serum creatinine of more than or equal to 50% (1.5-fold from baseline), or a reduction in urine output (documented oliguria of less than 0.5 ml/kg per hour for more than six hours) [14]. Continuous Renal Replacement Therapy (CRRT) in the course of AKI was initiated when indicated for pulmonary edema, oliguria (defined as urine output <0.5 mL/kg body weight per hour for >6 h), metabolic acidosis or hyperkalaemia not responding to conventional treatment, or uraemia (defined as urea nitrogen of >100 mg/dL). CRRT was available 24 hours a day, and no patient requiring CRRT was denied treatment due to likely futility.

### Statistical analysis

Discrete variables are expressed as counts (percentage) and continuous variables as means ± standard deviation (SD) or medians with the 25th to 75th interquartile range (IQR). For the demographic and clinical characteristics of the patients, differences between groups were assessed using the chi-squared test or Fisher's exact test for categorical variables and the Student's *t*-test or Mann-Whitney U test for continuous variables when appropriate. Multivariable stepwise logistic regression analysis was used to assess the impact of explanatory variables in outcome (ICU mortality) for both the pandemic and post-pandemic periods. In order to avoid spurious associations, variables entered in the logistic regression models were those with a relationship in univariate analysis (*P *≤0.1) or a potential plausible relationship with the outcome. To assess the possibility of a different impact of the explanatory variables in outcome for each period, interactions between explanatory variables and periods were used in the models. Results are presented as odds ratio (OR), 95% confidence intervals (CI) and *P*-values. Data analyses were performed using SPSS 16.0 for Windows (SPSS, Chicago, IL, USA).

## Results

Nine hundred and ninety-seven patients with completed outcomes admitted to ICU with confirmed Influenza (H1N1)v infection were analyzed in this study. Of these, 648 patients were affected during the 2009 pandemic Influenza (H1N1)v infection period and 349 patients during the post-pandemic Influenza (H1N1)v infection period.

**Figure 1:**
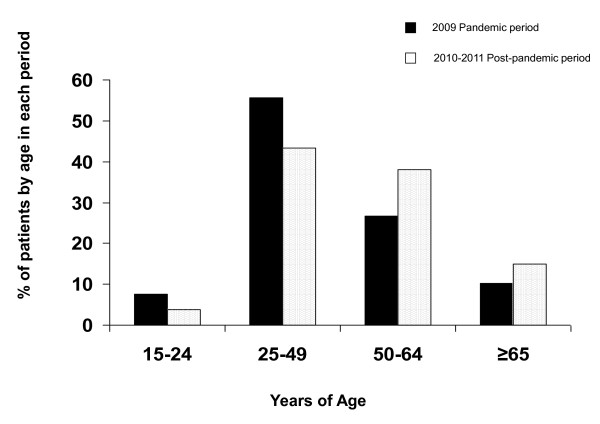
**Pandemic and post-pandemic age-specific influenza ICU admission**.

Patients from the post-pandemic Influenza (H1N1)v infection period were older, more frequently male and presented with more comorbidities than those affected by the 2009 pandemic Influenza (H1N1)v infection. Comorbidities included chronic obstructive pulmonary disease (COPD), chronic renal disease and hematological disease. Pandemic and post-pandemic age-specific influenza ICU admission baseline characteristics are displayed in Figure 1. Vaccination was only available in Spain during the post-pandemic period. A total of 19 (5.4%) received vaccination. Vaccination was more frequent in patients who presented with comorbidities than those who did not (18 (7.0%) vs. 1 (1.1%), *P *= 0.02). There was no difference in time to diagnosis between vaccinated and non-vaccinated patient groups (6 IQR (4 to 8) vs. 8 IQR (5 to 11), *P = *0.1. No particular comorbid condition was predominant in those vaccinated. Additional demographic data and clinical characteristics regarding delay in diagnosis and vaccination status are displayed in Table 1.

**Table 1:** Comparison of baseline characteristics between 2009 pandemic and post-pandemic Influenza (H1N1)v infection period

Variables	Pandemic period N = 648	Post-pandemic period N = 349	*P-value*
**Age. years**			
**Mean (SD)**	44.7 +/- 14.6	49.9 +/- 14.2	<0.001
**Median (IQR)**	34 (44 to 54)	40 (51 to 59)	<0.001
**Male (sex) n (%)**	374 (57.7)	237 (67.9)	0.01
**COPD n (%)**	108 (16.7)	78 (22.3)	0.02
**Asthma n (%)**	81 (12.5)	28 (8.0)	0.03
**Chronic heart failure n (%)**	47 (7.3)	36 (10.3)	0.09
**Chronic renal disease n (%)**	33 (5.1)	30 (8.6)	0.03
**Diabetes mellitus n (%)**	82 (12.7)	55 (15.8)	0.1
**Pregnancy n (%)**	31 (4.8)	13 (3.7)	0.4
**Obesity n (%)**	138 (21.3)	64 (18.3)	0.2
**Autoimmune disorders n (%)**	18 (3.4)	2(1.8)	0.5
**Hematological disease n (%)**	40(6.2)	37 (10.6)	0.01
**Neuromuscular disease n (%)**	24 (3.7)	4 (1.1)	0.02
**HIV infection n (%)**	13 (2.0)	13 (3.7)	0.1

Abbreviations: CHF, chronic heart failure; COPD, chronic obstructive pulmonary disease; CRF, chronic renal failure; DM, diabetes mellitus; HIV, human immunodeficiency virus; NM, neuromuscular

Acute Physiology And Chronic Health Evaluation (APACHE II) and Sequential Organ Failure Assessment (SOFA) scores were higher in post-pandemic Influenza (H1N1)v infection patients. In addition, this cohort of patients was admitted later to the hospital from time of symptom onset (4.2 +/- 2.6 vs. 4.8 +/- 3.4, *P *= 0.004) and had a longer delay from time of hospitalization to diagnosis (2.3 +/- 2.1 vs.6.5 +/- 3.8, *P *<0.001). Primary viral pneumonia was documented in 688 (69.0%) patients with no significant difference between the two periods. In 176 (17.7%) cases another pathogen was isolated at ICU admission and these patients were considered to have CARC.

Empiric antibiotic therapy was administered in all patients in accordance with local protocols. Post-pandemic Influenza (H1N1)v infection patients received empiric antiviral treatment less frequently (99.1% vs. 97.4%, *P *= 0.04), lower doses of oseltamivir (150 mg/day) (73.5% vs. 51.5%, *P *<0.001), delayed antiviral administration (5.6 +/- 3.5 vs. 4.7 +/- 2.9 days, *P *<0.001), and had higher use of zanamivir (7.2% vs. 0.6%, *P *<0.001) than those from the 2009 pandemic Influenza (H1N1)v infection period.

Comparison of complications during ICU stay was different between the two periods. Septic shock was present more frequently in the post-pandemic Influenza (H1N1)v infection period (54.4% vs. 45.4%, *P *= 0.007). Patients affected during the post-pandemic Influenza (H1N1)v infection period received mechanical ventilation (invasive or non-invasive) more frequently (80.8% vs. 71.8%, *P *= 0.002); however, there was no significant difference in implementation of invasive mechanical ventilation. Extracorporeal membrane oxygenation (ECMO) (n = 9) was more frequently implemented during the post-pandemic Influenza (H1N1)v infection period (1.7% vs. 0.5%, *P *= 0.07). AKI was more frequently identified during the post-pandemic Influenza (H1N1)v infection period (27.2% vs. 20.1%, *P *= 0.01) with the use of CRRT significantly different between the two periods. Further details are displayed in Table 2.

**Table 2:** Comparison of clinical presentation and therapy provided between 2009 pandemic and post-pandemic Influenza (H1N1)v infection period.

Variables	Pandemic period N = 648	Post-pandemic period N = 349	*P-value*
**APACHE II score**. **Mean (SD)**	13.9 +/- 7.2	16.3 +/- 7.7	<0.001
**SOFA score**. **Mean (SD)**	5.6 +/- 3.6	6.2 +/- 4.1	0.04
**Clinical presentation**			
**Primary viral pneumonia**	451 (69.6)	237 (67.9)	0.5
**COPD exacerbation**	36 (5.6)	24 (6.9)	0.4
**Community acquired respiratory co-infection**	107 (16.5)	69 (19.8)	0.2
**Secondary respiratory infections**	57 (9.3)	16 (4.7)	0.006
**Days from symptoms onset to hospital admission, Mean (SD)**	4.2 +/- 2.6	4.8 +/- 3.4	0.004
**Days from hospitalization to diagnosis, Mean (SD)**	2.3 +/- 2.1	6.5 +/- 3.8	<0.001
**Clinical failure**			
**Septic shock**	294 (45.4)	190 (54.4)	0.006
**MODS**	405 (62.5)	199 (57)	0.09
**Mechanical ventilation**	465 (71.8)	282 (80.8)	<0.001
**Invasive mechanical ventilation**	398 (61.4)	233 (66.8)	0.09
**Non-invasive mechanical ventilation**	163 (25.2)	115 (33)	<0.001
**Prone positioning**	95 (14.7)	67 (19.2)	0.06
**Septic shock**	294 (45.4)	190 (54.4)	0.007
**Acute kidney Injury**	130 (20.1)	95 (27.2)	<0.001
**Continuous renal replacement therapy**	53 (8.2)	45 (12.9)	<0.001
**Therapy administered**			
**Empirical oseltamivir**	631 (99.1)	339 (97.4)	0.04
**Days from symptoms onset to oseltamivir administration**	4.7 (2.9)	5.6 (3.5)	<0.001
**Zanamivir**	4 (0.6)	25 (7.2)	<0.001
**ICU mortality**	141 (21.8)	105 (30.1)	0.004

Abbreviations: ICU, intensive care unit; MODS, multiple organ dysfunction syndrome

In total, 246 (24.7%) patients died. No statistical difference in survival was observed between patient groups with respect to vaccination status (6 (5.7%) vs. 13 (5.3%), *P = *0.8). The median age for non-survivors was significantly higher in the post-pandemic period (42 years (54 to 61.5) vs. 36 years (IQR 47 to 61), *P *<0.001)). Mortality was significantly higher in the post-pandemic Influenza (H1N1)v infection period compared to the 2009 pandemic (H1N1)v infection period (105 (30.1%) vs. 141(21.8%) OR 1.5 (95% CI 1.1 to 2.1)). The baseline characteristics of patients in both groups who did not survive are compared in Table 3. Briefly, during the 2009 pandemic (H1N1)v infection period, APACHE II score, SOFA score, age, congestive heart failure, chronic renal disease, hematological disease, CARC, septic shock, MODS, invasive mechanical ventilation (MV) and CRRT were variables associated with ICU mortality (univariate analysis). APACHE II score, SOFA score, hematological disease, HIV, CARC, septic shock, MODS, invasive MV and CRRT were associated variables during the post-pandemic Influenza (H1N1)v infection period. Multivariate analysis (Table 4) confirmed that among patients in both groups the presence of hematological disease, the use of invasive MV, and CRRT were factors independently associated with a worse outcome. HIV was the only new variable independently associated with higher ICU mortality during the post-pandemic influenza (H1N1)v infection period. In addition, the only interaction statistically significant was between the presence of HIV and the post-pandemic Influenza (H1N1)v infection period.

**Table 3:** Risk factors for ICU mortality for 2009 pandemic and post-pandemic Influenza (H1N1)v infection patients.

Variable	Pandemic period N = 648			Post-pandemic period N = 349		
**Risk factor**	**Alive** **(n = 507)**	**Death** **(n = 141)**	***P-*value**	**Alive** **(n = 244)**	**Death** **(n = 105)**	***P-v*alue**
SOFA	4.9 (3.0)	8.4 (4.3)	<0.001	5.1 (3.5)	8.6 (4.1)	<0.001
APACHE	12.53 (6.1)	19.69 (8.3)	<0.001	14.11 (6.1)	21.2 (8.5)	<0.001
**Demographics**						
Age. Mean (SD)	43.72 (14.0)	48.4 (16.3)	<0.001	49.0 (14.1)	52.0 (14.3)	0.07
Male sex	285 (56.2)	89 (63.1)	0.14	162 (66.4)	75 (71.4)	0.35
**Comorbidities**						
**≥1 underlying medical conditions**	353 (69.6)	112 (79.4)	0.02	177 (72.5)	81 (77.1)	0.3
COPD	87 (17.2)	21(14.9)	0.52	52 (21.3)	26 (24.8)	0.47
Asthma	69 (13.6)	12 (8.5)	0.105	23 (9.4)	5 (4.8)	0.14
Chronic heart failure	31(6.1)	16(11.3)	0.03	23(9.4)	13(12.4)	0.41
Chronic renal disease	19 (3.7)	14 (9.9)	0.003	18 (7.4)	12 (11.4)	0.21
Diabetes mellitus	62 (12.2)	20 (14.2)	0.53	41(16.8)	14 (13.3)	0.41
Obesity	104 (20.5)	34 (24.1)	0.35	50 (20.5)	14 (13.3)	0.11
Autoimmune disease	13 (2.6)	8 (5.7)	0.06	7 (2.9)	7 (6.7)	0.09
Hematologic disease	19 (3.7)	21 (14.9)	<0.001	14 (5.7)	23 (21.9)	<0.001
Neuromuscular disease	18 (3.6)	6 (4.3)	0.69	2 (0.8)	2 (1.9)	0.38
HIV infection	9 (1.8)	4 (2.8)	0.42	2 (0.8)	11 (10.5)	<0.001
Pregnancy	24 (4.7)	7 (5)	0.9	11 (4.5)	2 (1.9)	0.2
Vaccination				13 (5.3)	6 (5.7)	0.8
**Clinical presentation**						
Viral pneumonia	355 (70)	96 (68.1)	0.6	164 (67.2)	73 (69.5)	0.6

COPD exacerbation	31 (6.1)	5 (3.5)	0.2	21 (8.6)	3 (2.9)	0.05
Community acquired respiratory co-infection	74 (14.6)	33 (23.4)	0.01	38 (15.6)	31 (29.5)	0.003
**Complications**						
Septic shock	190 (37.5)	104 (73.8)	<0.001	106 (43.4)	84 (80)	<0.001
MODS	282 (55.6)	123 (87.2)	<0.001	111 (45.5)	88 (83.8)	<0.001
Acute kidney injury	69 13.6)	61 (43.3)	<0.001	43 (17.6)	52 (49.5)	<0.001
Therapy administered						
Empirical oseltamivir	497(99.6)	134 (97.1)	0.002	238 (97.9)	101 (96.2)	0.1
Continuous renal replacement therapy	15 (3.0)	38 (27.0)	<0.001	12 (4.9)	33 (31.4)	<0.001
Mechanical ventilation	327 (64.5)	138 (97.9)	<0.001	181 (74.2)	101 (96.2)	<0.001
Invasive mechanical ventilation	263(51.9)	135(95.7)	<0.001	135(55.3)	98(93.3)	<0.001
Non-invasive mechanical ventilation	125(24.7)	38(27.3)	0.5	86(35.7)	29(27.6)	0.1

Abbreviations: CARC, community acquired respiratory co-infection; COPD, chronic obstructive pulmonary disease; HIV, human immunodeficiency virus; ICU, intensive care unit; MODS, multiple organ dysfunction syndrome

**Table 4:** Multivariate analysis for risk factors associated with ICU mortality during the 2009 pandemic and post-pandemic Influenza (H1N1)v infection period.

Variable	OR	95% CI	*P*-value
APACHE II score	1.076	1.040 to 1.114	<0.001
Hematologic disease	3.506	1.747 to 7.038	<0.001
Continuous renal replacement therapy	5.812	3.101 to 10.893	<0.001
Invasive mechanical ventilation	6.890	3.538 to 13.418	<0.001
HIV infection in pandemic Influenza (H1N1)v infection period *	1.362	0.279 to 6.648	0.702
HIV infection in post-pandemic Influenza (H1N1)v infection period *	19.835	2.236 to 175.954	0.007

The interaction HIV infection with period was statistically significant (*P*-value = 0.05).

Abbreviations: HIV, human immunodeficiency virus; ICU, intensive care unit

## Discussion

Data from this study evidenced that the post-pandemic Influenza (H1N1)v infection period targeted patients with a worse basal condition than those of the 2009 pandemic (H1N1)v infection period. Patients from the post-pandemic period were older, had more comorbidities, a more advanced clinical presentation on admission, higher severity scores, increased incidence of CARC and septic shock, and an increased requirement for mechanical ventilation than those from the pandemic period. In addition, patients from the post-pandemic period had a higher mortality rate.

Experiences during the post-pandemic Influenza (H1N1)v infection period have demonstrated that the 2009 pandemic (H1N1) virus is still circulating and, importantly, still causing severe disease in younger people [10,15-17]. During the immediate post-pandemic period multiple subtypes of influenza virus were analyzed in order to evaluate the isolation of other types of influenza viruses. The Ministry of Health reported that 98% of isolates were Influenza A (0.02% AH1; 0.08% AH1N1; 0.09 AH3; 0.33% AH3N2 and 99.5% 2009AH1N1), 1.4% Influenza B and 0.1% Influenza C [16].

Current World Health Organization (WHO) guidance strongly recommends the use of oseltamivir for severe or progressive infection with 2009 pandemic (H1N1)v infection, with zanamivir as an alternative if the infecting virus is oseltamivir-resistant [17]. Although the majority of 2009 pandemic (H1N1) viruses are susceptible to oseltamivir and very little resistance to oseltamivir has been found to date [18], the use of zanamivir has been 12 times more frequent during the post-pandemic Influenza (H1N1)v infection period. Previous studies [3,4] have shown that early oseltamivir administration resulted in increased survival among critically ill, ventilated patients and reduced complications in patients hospitalized during the 2009 pandemic (H1N1)v infection period. In addition, oseltamivir suppresses viral load more effectively when given early [19,20]. It is important to recognize that the early control of viral load in patients may be explained by the actions of innate immunity followed by early anamnestic adaptive immune response [21]. The lack of an appropriate and early immune response because of innate humoral or cellular immunodeficiencies, concomitant use of immunosuppressors, or transient immunoparesis due to severe concomitant co-infection may predispose some patients to develop severe clinical progression [22]. The incidence of HIV was 2% and 3.7% during the 2009 pandemic (H1N1)v infection period and post-pandemic Influenza (H1N1)v infection period respectively, but, interestingly, HIV was the only independent additional factor associated with higher ICU mortality in the post pandemic period. As previously reported, HIV patients, well controlled on Highly Active Antiretroviral Therapy (HAART), had a similar clinical outcome and prognosis to that of non-HIV patients during the pandemic period [23]]. However, in our study, HIV appeared to be a new independent risk factor for worse outcome. The immune response associated with severe viral infections remains unclear in HIV patients and needs to be further investigated, as the virus was no longer acting in a naive host.

While in 2009 the new virus found large numbers of people with no previous immunity against it, in 2010-2011 the situation dramatically changed. Large numbers of people had already received the vaccine, or alternatively had acquired natural immunity following infection by the time that the 2010-2011 season started. This created a more difficult environment for the virus to thrive, with a decreased percentage of susceptible individuals. It is plausible that with these changes in the population the virus would preferentially target hosts with chronic comorbidities, such as COPD, diabetes or HIV patients with altered cellular immunity. These conditions could potentially impair the efficiency of the immune response in clearing the infection. Proportional changes of the susceptible population could explain the observed differences in the profile of critically ill patients infected and the increased severity at admission to ICU. This trend is closer to the "normal" behaviour of seasonal influenza.

One important feature in both phases was the presence of acute respiratory failure as reflected by the need for invasive mechanical ventilation as an independent predictor of death. This finding is compatible with viral pneumonia being the principal cause of death in these patients and reinforces the role of the virus in causing critical illness. Despite the fact that non-invasive mechanical ventilation was not recommended for the management of patients affected by H1N1, its use was observed in 33% of cases in the post-pandemic Influenza (H1N1)v infection period. This finding may explain the delay in onset of invasive mechanical ventilation and a subsequently worse outcome during this period. It is important to recognise that the presence of neither CARC nor septic shock were independently associated with ICU mortality.

The presence of CARC has been a topic commonly discussed during previous pandemics [7]. A substantial base of laboratory evidence for synergism between Influenza A and bacterial microorganisms has been suggested [24]. During the 1918-1919 Influenza A pandemic, most deaths were attributed to concurrent bacterial infection [25]. Histopathological samples of lung tissue sections from fatal 1918 influenza case materials frequently revealed histopathologic findings consistent with acute bacterial pneumonia [26]. Nevertheless, this data result is surprising since CARC did not represent an independent risk factor for ICU mortality during either the 2009 pandemic (H1N1)v infection period or during the post-pandemic Influenza (H1N1)v infection period. While many studies [27] have demonstrated temporal relationships between influenza activity and CARC, and also an increasing number of patients affected by CARC during the post-pandemic period, the present study did not show significant differences between the two periods.

During the first case reports of the 2009 pandemic (H1N1)v infection period, impairment of renal function was commonly observed and patients who died had documented multiple organ failure with significantly higher rates of renal failure [28]. AKI is a complex disorder that has been observed to occur in ICU patients with severe presentation of illness during the 2009 pandemic (H1N1)v infection period [29], and it has been associated with increased mortality rates. Interestingly, the rate of AKI was significantly higher during the post-pandemic Influenza (H1N1)v infection period (27.2% vs. 20.1%, *P *<0.001) and, as has been reported previously [9], is associated with an increase in ICU mortality during the post-pandemic period. The cause-and-effect relationship between viral infection and kidney injury is not clear, nor is the reason for an increased incidence in the post-pandemic period. Glomerular deposition of viral antigen, cytokine dysregulation associated with severe viral infection, and virus-related rhabdomyolysis might partially explain the pathophysiology of AKI. However, there are several factors that might contribute to an increased incidence during the post-pandemic period. First, patients admitted in the post-pandemic period were older and presented with a higher severity of illness. Second, patients admitted in the post-pandemic period had a longer delay between diagnosis and onset for antiviral therapy. Finally, a higher incidence of septic shock might contribute to an increase in AKI. The reasons for and implications of AKI development in patients with H1N1 have not been clearly elucidated, and further research is needed in order to allow early identification of the subject at risk and provide targeted medical care.

The present study has some limitations that should be addressed. First, only adults admitted to Spanish ICUs were included and, therefore, our results might not be generalized to other countries or children. However, our study included more than 70% of all patients admitted to the ICU during the pandemic and post-pandemic Influenza (H1N1)v infection periods. Management of patients was not standardized and management practices were chosen in accordance with local protocols. Nevertheless, it has the strength of a prospective, multi-center design with a large number of patients. ECMO was rarely implemented in Spain (n = 9, 0.9%) and, therefore, no conclusions can be reached. Second, patients presenting in the post-pandemic Influenza (H1N1)v infection period had a delay in diagnosis, but time to diagnosis might be a non-objective parameter that depends on patient experience, the grade of physician and index of suspicion. Moreover, the unavailability of daily RT-PCR in some institutions during the post-pandemic period could prolong the time to diagnosis. Finally, the CD4 count was not recorded in HIV patients. Based on the epidemiological nature of the study only patients diagnosed with HIV were included in the group. It is important to note that some patients develop a low CD4 count when critically ill [30]. In addition, low CD4 cell counts, time since HIV diagnosis, HAART treatment or AIDS stage of disease; conversely, have not been identified as risk factors for ICU mortality in the post-HAART era [31]. Therefore, CD4 count in HIV patients should be further investigated. Finally, the marked change in time to diagnosis also suggests that there was laxity in testing during the present study. Cases may have been missed in the post-pandemic period with selection bias contributing to observed differences.

## Conclusions

In summary, pandemics, like the viruses that cause them, are unpredictable. So was the immediate post-pandemic Influenza (H1N1)v infection period. The main findings were that the mortality rate and severity scores were higher in the post-pandemic period. A lower clinical awareness and a more vulnerable population may explain the worse outcome in the post-pandemic period. The development of protocols, educational programs and prevention recommendations need to be implemented to avoid further diagnosis and treatment delays. As was previously warned [10], physicians should maintain a high level of clinical suspicion for future waves in order to prevent the unexpected increase in mortality observed in this study.

## Key messages

• The mortality rate and severity scores were higher in the post-pandemic Influenza (H1N1)v infection period.

• During the post-pandemic Influenza (H1N1)v infection period, patients were older, with more comorbidities and with delayed hospital admission.

• A more vulnerable population was affected in the post-pandemic Influenza (H1N1)v infection period.

• HIV was the only new variable independently associated with higher ICU mortality during the post-pandemic Influenza (H1N1)v infection period.

## Abbreviations

AKI: acute kidney injury; AKIN: Acute Kidney Injury Network: APACHE II: Acute Physiology and Chronic Health Evaluation II; BMI: body mass index; CAP: community-acquired pneumonia; CARC: Community Acquired Respiratory Co-Infection; CDC: Centers for Disease Control and Prevention; CI: confidence interval; CIBERES: Spanish Biomedical Research Center Network on Respiratory Diseases; CK: creatinine kinase; COPD: chronic obstructive pulmonary disease; CRRT: continuous renal replacement therapy; GFR: glomerular filtration rate; HAART: Highly Active Antiretroviral Therapy; HIV: human immunodeficiency virus; OR: odds ratio; HAP: hospital-acquired pneumonia; HCAP: healthcare-associated pneumonia; ICU: intensive care unit; IQR: interquartile range; MODS: Multiple Organ Dysfunction Score; MV: mechanical ventilation; OR: odds ratio; REIPI: Spanish Network for Research on Infectious Disease; RRT: renal replacement therapy; RT-PCR: real-time polymerase chain reaction; SD: standard deviation; SEMICYUC: Spanish Society of Intensive Care Medicine; SOFA: Sequential Organ Failure Assessment; WHO: World Health Organization.

## Competing interests

The authors declare that they have no competing interests

## Authors' contributions

IML made a substantial contribution. AR and IML assisted in the design of the study, coordinated patient recruitment, analyzed and interpreted the data and assisted in writing the paper. LV, CL, RG, JI, JEG, JB, AA and IN made important contributions to the acquisition and analysis of data. AT and JFBM were involved in revising the manuscript critically for important intellectual content. IML, AR, DS and ED made substantial contributions to the conception, design, analysis and interpretation of data and revised the final manuscript version. All authors read and approved the final manuscript.
